# Gallbladder Agenesis and Cystic Duct Absence in an Adult Patient Diagnosed by Magnetic Resonance Cholangiography: Report of a Case and Review of the Literature

**DOI:** 10.1155/2009/674768

**Published:** 2010-02-01

**Authors:** Valeria Fiaschetti, Giovanna Calabrese, Silvia Viarani, Gabriele Bazzocchi, Giovanni Simonetti

**Affiliations:** Department of Diagnostic and Molecular Imaging, Interventional Radiology, Nuclear Medicine and Radiation Therapy, University of Rome “Tor Vergata”, 81 Oxford street, 00133 Rome, Italy

## Abstract

Gallbladder agenesis (GA) is a rare congenital anomaly of the biliary system often associated with other congenital abnormalities. Patients become symptomatic in 23% of cases. GA is often misinterpreted as other diseases, therefore, leading to unnecessary surgery. We report a case of congenital GA associated to cystic duct absence and a biliary tract abnormality diagnosed by Magnetic Resonance with Cholangiopancreatography.

## 1. Introduction

Gallbladder agenesis (GA) is a rare congenital anomaly of the biliary system. Reported for the first time in human beings by Bergman back in 1702 [1], it has since been described several times in case reports.

The etiology of GA is unknown, it is often a sporadic occurrence with no clear causes. However, there are families in which the condition has occurred in several members, suggesting that there are familial hereditary forms of GA [2, 3].

The agenesis is attributed to an abnormality in the embryonic development, so most cases of gallbladder agenesis are associated with other congenital abnormalities, including those of the bile system [4]. It is present in 1/6 of cases of biliary atresia; the isolated absence of the gallbladder and cystic duct is rare [5]. The average incidence of gallbladder agenesis at birth is around 0.02% (A6); it occurs without sex-linked traits and with variable penetrance [6]. 

Patients become symptomatic in 23% of cases, and AG will almost always be misinterpreted as cholecystitis with cystic duct obstruction or as sclero-atrophic gallbladder, therefore, leading to unnecessary surgery [7]. 

It is difficult to establish a correct preoperative diagnosis of GA in symptomatic patients because of the nonspecific nature of the symptoms.

We report a case of congenital GA in an adult patient, associated to cystic duct absence and a biliary tract abnormality diagnosed by Magnetic Resonance with Cholangiopancreatography (MRCP). We also discuss the etiology and physiopathology of this abnormality and diagnostic tools employed.

In our knowledge this is the first case of GA preoperatively correctly diagnosed by radiological imaging, in particular MRCP, described in the literature.

## 2. Case Report

A 44-year-old man was admitted to our attention for upper abdominal pain, bloating, and dyspepsia for the last 6 months. His blood pressure and pulse rate were regular and his body temperature was 36.7°C. Results of all hematological and biochemical investigations were within normal limits. 

**Figure 1:**
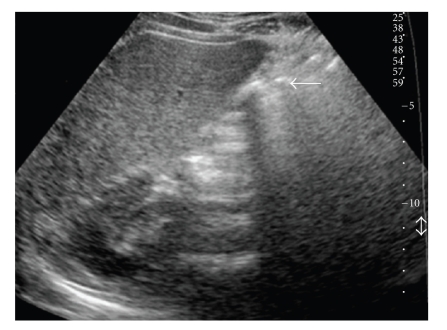
Ultrasonography scan demonstrates an hyperechoic area with an acoustic shadow in the gallbladder fosse (arrow). These findings usually are suspected for sclero-atrophic or contracted lithiasic gallbladder.

Ultrasonography (US) examination did not visualize the gallbladder clearly. However, it demonstrated a hyperechoic area with an acoustic shadow in the gallbladder fossa, and this finding was suspected for sclero-atrophic to be contracted lithiasic gallbladder (Figure 1).

MRCP was subsequently performed with a 1.5 Tesla magnet (Philips Gyroscan Intera; Best, Medical Systems, Netherlands), equipped with a Master dynamic gradient system (30 mTm maximum power and 150 mTm/msec slew rate) using a phasedarray body coil. 

The patient underwent the MRCP study after fasting for 8 hours.

**Figure 2:**
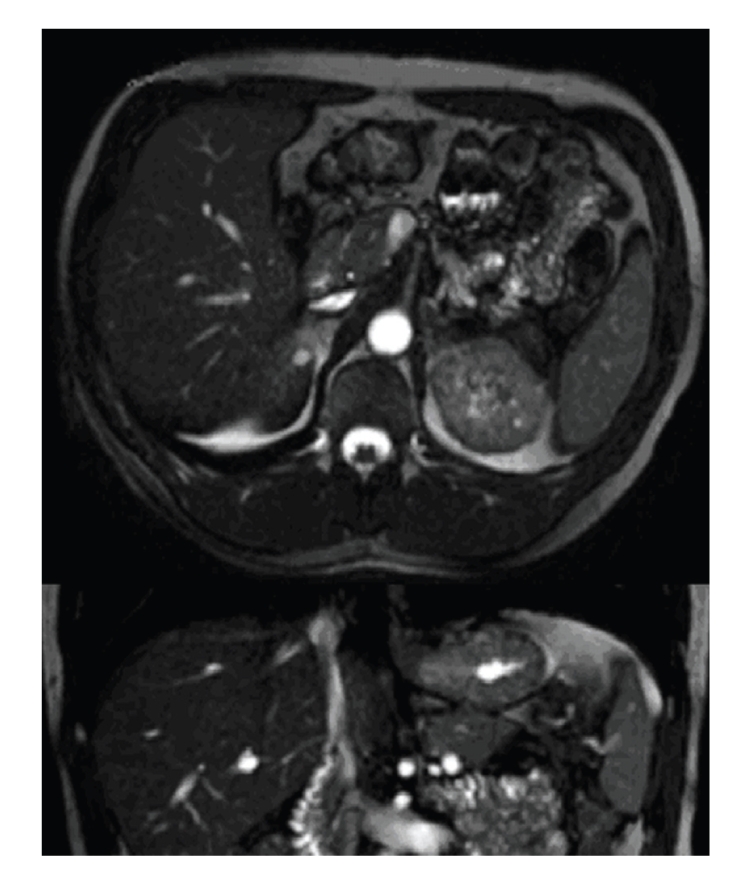
Transverse T2-weighted TSE sequences (up) and Coronal BALANCE sequences (down) do not visualize the gallbladder in the cholecystic fossa.

**Figure 3:**
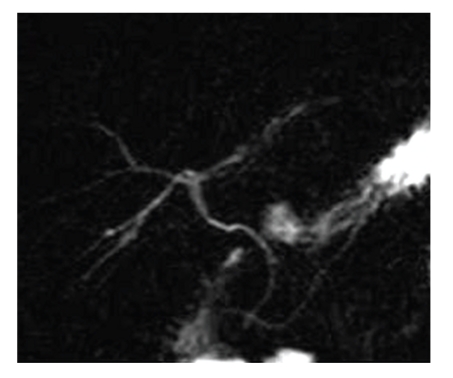
3D-MIP breath-hold acquisition of a single slice in the coronal plane demonstrates the absence of both the gallbladder and the cystic duct or an ectopic gallbladder. The intrahepatic bile ducts appear normal with no images of stenosis or repletion defects. The hepatocholedocho is visualized until the second part of the duodenum, in correspondence to the Major papilla. Also the Wirsung duct demonstrates an anatomical variation; it is visualized until the initial part of the duodenum, in correspondence to the Minor papilla.

The examination protocol consisted of an axial T1-weighted 2D FLASH and axial T2-weighted TSE sequences and axial SPIR to localize the acquisition volume for the MR Cholangiography sequences. We performed BALANCE sequences to obtain an accurate anatomical resolution and gallbladder fossa visualization. The MRCP study consisted of a 3D-MIP breath-hold acquisition of a single slice in the coronal plane, positioned so as to obtain complete visualization of the intra- and extrahepatic biliary tract, with a single-slab RARE sequence (Figures 2 and 3).

The MRCP showed the absence of both the gallbladder and the cystic duct. There were no images of cystic lesions in the intrahepatic area, in the lesser omentum, in the retroperitoneal and retrohepatic areas, within the falciform ligament, or in the retroduodenal area compatible with condition of ectopic gallbladder (Figure 3). 

The intrahepatic bile ducts appeared normal with no images of stenosis or repletion defects. 

Moreover, MRCP demonstrated an anatomical variation of choledochopancreatic duct junction. In fact the bile duct was visualized up until the second part of the duodenum, in correspondence of the Major papilla. The Wirsung duct, on the other hand, was visualized until the initial part of the duodenum, in correspondence of the Minor papilla. 

Clinical history revealed familial gallbladder agenesis diagnosticated into 2 paternal aunts during laparoscopic surgery. 

The patient underwent medical treatment with complete control of symptoms.

## 3. Discussion

Anatomic anomalies of the biliary tract are not uncommon, but gallbladder and cystic duct agenesis is rare [8]; it is often discovered incidentally and is usually asymptomatic [9]. GA can be observed in both children and adults, with a mean age of 46 years at the time of the diagnosis [10]. It is often a casual finding during abdominal surgery or at autopsy. 

The prevalence range is 0.007–0.13%. The incidence of this malformation is slightly lower in surgical cholecystectomy series (0.007–0.027%) than that in autopsy reports [11].

GA diagnosed during surgery has a female predominance of 3 : 1, while cases found in autopsies have an equal sex ratio [12]. 

The gallbladder develops from the caudal part of the hepatic diverticulum in the fourth week of prenatal life. There are two theories regarding nondevelopment of the gallbladder [13, 14]. According to one theory, the hepatic diverticular bud of the foregut fails to develop properly into the gallbladder and cystic duct. The other theory holds that, following solid-phase development, there is a failure of recanalization of the cystic duct and gallbladder. Isolated GA results when the cystic bud does not develop [15]. Accordingly, GA usually occurs together with cardiovascular and gastrointestinal abnormalities, because the cystic bud growth disrupts development between the sinus venus cordis and the paired omphaloenteric and umbilical veins [16].

GA occurs alone in 70–82% of cases (31.6% asymptomatic cases and 55.6% symptomatic cases). It occurs in association with additional malformations in the remaining 12.8–30% of cases, that fall into two subgroups: one with atresia of the bile ducts or choledochal cyst (9%), and the other with normal bile ducts but with distant multiple fetal anomalies (12.8–21%) [17–19].

In newborns, AG is associated with one or more defects, sometimes incompatible with life. GA has been reported to be associated with many other gastrointestinal, skeletal, cardiovascular, and genito-urinary malformations, such as ventricular septal defect, imperforate anus, duodenal atresia, malrotation of the gut, pancreas divisum, hypoplasia of the right hepatic lobe, duplication cysts of the hepatic flexure, renal agenesis, undescended testes, and syndactyly [20].

GA has no characteristic symptomatology, patients become symptomatic in about 23% of cases. Right upper quadrant abdominal pain is present in 90% of the cases, nausea and vomiting in 66%, fatty food intolerance in 37%, dyspepsia in 30%, and jaundice in 35% [21]. The jaundice is due to associated choledocholithiasis with or without ascending cholangitis [9, 22]. Most of the adult patients with GA are asymptomatic. The symptoms may be secondary to concomitant biliary pathologies such as primary duct stones and biliary dyskinesia (patients may have a congenital abnormality of function in the form of a significant higher sphincter of Oddi resting pressure and an increase in the proportion of retrograde propagation of phasic muscular contraction with regurgitation of pancreatic or duodenal contents), or it may be related to nonbiliary causes such as esophagitis and duodenitis [20].

Reviewing the literature, we noticed that, with the exception of two cases of GA, symptomatic patients are still unnecessarily operated on because the preoperative investigations carried out failed to demonstrate the exact diagnosis [23, 24]. 

GA represents a difficulty for the surgeon [25, 26]: during laparoscopic surgery, the biliary or portal structures can easily be wounded during dissection as one searches for a gallbladder that does not exist. 

If the diagnosis of GA is made during operation, the surgeon must prove GA by thoroughly examining the most common sites for ectopic gallbladder, which are intrahepatic, retrohepatic, on the left side, or within the leaves of the lesser omentum or within the falciform ligament, retroduodenal, retropancreatic, and retroperitoneal [14] . The absence of normal anatomical structures and the inability to pull on the gallbladder to dissect the triangle of Callot represent a risk of iatrogenic injury, and it is the most common cause of conversion from a laparoscopic procedure to a traditional open laparotomy [27].

Ultrasonography is actually the investigation method of choice for the diagnosis of common bile duct stones, with a sensitivity of 95–98%. Crade et al., defined three categories of abnormal ultrasounds of the gallbladder: shadowy gravity-dependent opacities within the gallbladder, nonvisualization of the gallbladder lumen, and nonshadowy opacities within the gallbladder lumen. The accuracy of US in these three different categories is 100%, 96%, and 61%, respectively [28]. The great difficulty in visualizing a contracted gallbladder on stones is well known. According to Hammond, there is always either a recognizable segment of wall or a thin rim of bile identifying the gallbladder [29].

However, the examination conditions as well as the examiner's experience do not always permit such accurate appreciation. Shadowy opacities misdiagnosed as stones can be due to intestinal gas artifacts or to other structures in close proximity, such as a calcified hepatic lesion or a surgical clip [30].

In our patient, the duodenum was probably misdiagnosed as sclero-atrophic or contracted lithiasic gallbladder.

Endoscopic retrograde cholangiopancreatography (ERCP) has been used in addition to other diagnostic methods [31]. However, it is associated with significant mortality and morbidity and with high rates of unsuccessful cannulation [32]. Moreover, the nonvisualization of the gallbladder is, regularly, interpreted as an occlusion of the cystic duct.

MRCP is a noninvasive and well-demonstrated imaging method in the evaluation of the biliary tract [33–35]. As it does not require contrast administration to visualize the bile, it is not compromised by biliary stasis. It can also demonstrate an excluded and/or ectopic gallbladder.

In our case, MRCP allowed to make the correct preoperatively diagnosis with a noninvasive examination, avoiding unnecessary surgical exploration, and minimizing the risk of complications. Moreover, it provided accurate anatomical details about the bile tree conformation excluding the condition of ectopic gallbladder too.

In conclusions, GA should be kept in mind whenever the gallbladder is improperly visualized in routine imaging methods in patients with biliary-type pain. 

MRCP technique may not yet replace ultrasound as the gold standard of acute gallbladder imaging but it revealed an ideal complementary study to inconclusive ultrasonographic studies. The correct preoperative diagnosis of GA is fundamental to avoid a needless surgical exploration, which might be risky.
